# Unconstitutionality of the income tax calculation when offsetting foreign taxes with regard to the exemption of the subsistence minimum? A quantitative analysis

**DOI:** 10.1016/j.heliyon.2024.e41096

**Published:** 2024-12-09

**Authors:** Thomas Kollruss

**Affiliations:** IU International University of Applied Sciences Germany, Erfurt; Open Access Publication Enabled By IU International University of Applied Sciences, Germany

**Keywords:** Income taxation, Constitutional law, Tax calculation, Foreign income, Imputation method, Tax policy

## Abstract

The present study addresses a previously unexamined question of whether the calculation of income tax in the case of foreign tax credits violates constitutional law. Methodologically, this is investigated via quantitative analysis. As part of a quantitative analysis it is shown that the current method of calculating income tax when offsetting foreign taxes violates constitutional law in the form of the subjective net principle, as the taxpayer loses part of the tax-effective basic allowance deduction due to the calculation method; thus, the minimum subsistence level is no longer fully exempt from taxation with income tax. To address this issue, this study proposes a quantitative approach to calculating income tax, which allows for the calculation of income tax in the country of residence in accordance with the constitution when foreign taxes are offset. This method effectively and completely exempts the taxpayer's minimum subsistence level from income taxation. States can use this approach as a guideline to organise their income tax calculation in line with constitutional law.

## 1 Introduction and research question

According to constitutional law (ability-to-pay principle, subjective net principle, welfare state principle) and settled case law, the income of the taxpayer—a natural person—in the minimum subsistence level is not taxable (German Federal Constitutional Court/BVerfG 2008, 2004, 1990 [3]). In this area, there is no taxable capacity on the part of the taxpayer, as he or she must spend income in the amount of the minimum subsistence level to cover his or her necessary living expenses (food, drink, clothing, housing, health). In income tax law, the nontaxation of the minimum subsistence level is standardised by the so-called basic tax-free allowance.^1^ The basic tax-free allowance represents income in the amount of the minimum subsistence level. As a result, of a taxpayer's total annual income, only the income above the basic tax-free allowance is subject to income tax. In a formula for calculating income tax rates, the basic tax-free allowance is therefore deducted (basic tax-free allowance deduction),^2^ and only income above the basic tax-free allowance is subject to taxation. Every taxpayer—regardless of the amount of total annual income—including millionaires—receives a basic tax-free allowance deduction of the same amount, which corresponds to the flat-rate minimum subsistence level of a person for a calendar year. According to constitutional law, the subjective net principle and case law, the taxpayer must be granted the full amount of the basic tax-free allowance (German Federal Constitutional Court/BVerfG 2008, 2004, 1990 [3]).

This study examines whether the calculation of income tax when offsetting foreign taxes (imputation method for foreign income; § 34c EStG) violates constitutional law in the form of the subjective net principle if, owing to its method and structure, the taxpayer partially loses the deduction of the basic tax allowance or if his taxable minimum subsistence level is not fully exempt from income tax.

The limiting effect of the tax calculation in this respect may result from the fact that the basic allowance deduction is partially offset against taxed foreign income so that it has no tax-reducing effect. If the taxpayer receives domestic and foreign income, the basic allowance is deducted from the taxpayer's total income when the income tax is calculated. The taxpayer's country of residence, which applies the imputation method, thus also partially offsets the basic tax-free amount against foreign income that has already been subject to income tax abroad and that is also subject to taxation in the country of residence via the imputation method. If the tax rate on foreign income in the foreign source state is higher or the same as the tax rate in the state of residence, no additional income tax is payable on foreign income in the state of residence under the imputation method (case 1). However, foreign income is taxed income, so the basic allowance deduction in the country of residence is applied to income that has already been taxed and therefore has no partial tax-reducing effect.

The same applies if the tax rate in the country of residence is higher than the tax rate in the foreign source country and if the additional tax in the country of residence, after offsetting the foreign tax on foreign income, is only partially offset by the basic tax allowance deduction or its tax-reducing effect (case 2).

However, the imputation method means that foreign income is treated as domestic income, as an equivalent tax burden to domestic income is assumed in the amount of the foreign tax that can actually be offset against the domestic tax of the country of residence.

The situation is different for domestic income that has already been subject to taxation or tax prepayment on income tax (e.g., wage tax, withholding taxes). Here, the exemption of the taxable minimum subsistence level from taxation with income tax or the full effect of the basic tax-free allowance is also guaranteed by the fact that an advance tax payment that exceeds the deduction of the tax effect of the basic tax-free allowance deduction or the annual income tax is fully refunded (§ 36 para. 2 no. 2 EStG). This ensures that the basic allowance deduction is granted in full or that its tax effect is guaranteed.

As part of the tax calculation when offsetting foreign taxes (§ 34c EStG), the taxpayer may lose part of the tax relief effect of the basic tax allowance deduction so that, contrary to constitutional law, income in the amount of the minimum subsistence level or the basic tax allowance is no longer exempt from income tax. This is analysed below for the first time via quantitative analysis. In the literature and research, this burdensome effect of tax calculation when foreign taxes are credited has not been recognised, let alone addressed. This study therefore contributes to the literature.

The effect of the partial loss of the tax-reducing effect of the basic allowance deduction through the tax calculation when offsetting foreign taxes is likely to be particularly pronounced if the foreign income is relatively high in relation to the taxpayer's domestic income or accounts for the majority of the taxpayer's total income (sum of domestic and foreign income) and if the foreign tax rate on foreign income is at least equal to or higher than the domestic tax rate.

Furthermore, this study develops proposals for the revision and constitutional design of the tax calculation, taking into account the crediting of foreign taxes, with a view to exempting the minimum subsistence level from taxation.

## 2 Literature review and contribution of the study to the literature

The question as to whether the calculation of income tax when offsetting foreign taxes violates constitutional law if the tax calculation method results in the taxpayer losing part of the tax-effective basic allowance deduction and thus no longer having their minimum subsistence level exempted from income tax has not yet been addressed or analysed in the literature. The same applies to the question of how the income tax calculation must be organised in line with constitutional law when offsetting foreign taxes so that the taxpayer's minimum subsistence level is fully exempt from income tax. There is not a single study available that addresses these questions. Therefore, no literature can be cited and analysed in this regard. The literature has not yet addressed these questions, which concern the fundamental architecture and design of a tax system against the background of constitutional law. These questions are fundamentally important for all states. This is because constitutional law, in the form of the ability-to-pay principle and the subjective net principle, obliges a state to be fully exempt from taxation the income of a taxpayer in the amount of the minimum subsistence level or the basic tax-free amount.

Levy (1959) [6] addresses the effect of personal income exemptions on the income tax structure. However, this study does not address the cross-border receipt of income or the method of calculating income tax when foreign taxes are credited, regardless of whether, in these cases, the existing method of tax calculation is suitable for fully exempting the taxpayer's minimum subsistence level from taxation.

Gerber et al. (2020) [2] discuss how the structure of the tax system affects its progressivity. The specific tax calculation methodology related to the deduction of the basic tax-free allowance and its impact on the exemption of the minimum subsistence level from taxation is not addressed. The constitutional requirements for the tax calculation are not addressed.

In its case law, the German Supreme Tax Court/BFH (2022) [5] and (2013) [4] assume that the limited creditability of foreign taxes in the country of residence does not violate constitutional law (ability-to-pay principle). Accordingly, the crediting of foreign taxes can be capped at the amount of domestic tax due on foreign income (the maximum crediting amount). However, the court has not yet examined or dealt with whether the method of calculating income tax when offsetting foreign taxes fulfils the subjective net principle with regard to the exemption of the taxpayer's minimum subsistence level from taxation, particularly when offsetting foreign taxes.

From the perspective of tax law, Englisch and Kube (2023) [1] address the constitutional obligation to exempt the taxpayer's minimum subsistence level from income tax. However, they do not address the necessary quantitative tax calculation methodology, nor do they address the question of what a constitutional taxation methodology should look like. They also do not address the question of whether the current tax calculation methodology complies with constitutional requirements. The authors do not address the case of offsetting foreign taxes and granting the tax subsistence minimum when calculating income tax.

Sarmento (2023) [8] deals generally with the crediting of foreign taxes in the country of residence to avoid double taxation when receiving foreign income. The necessary structuring of the income tax calculation, taking into account constitutional law and the exemption of the minimum subsistence level from taxation, is not addressed.

Sabirov (2024) [7] deals with the definition and general consideration of the subsistence minimum in income taxation. The study does not address the question of how the minimum subsistence level should be taken into account in income tax calculations in a manner that is consistent with constitutional requirements.

This study is the first to address the question of whether the calculation of income tax when offsetting foreign taxes is compatible with the constitutional requirements of the exemption of the subsistence minimum. A corresponding investigation and quantitative analysis have not yet been carried out. Furthermore, the study answers the previously unaddressed question of how an income tax system with foreign tax credits must be specifically designed with respect to the method of calculating the tax so that it complies with the constitutional requirements and effectively exempts the minimum subsistence level or income in the amount of the basic tax-free allowance from taxation. Therefore, this study significantly expands the state of knowledge and the literature. It also solves an important problem, namely, how the tax system should be organised in concrete terms when foreign income is received including the imputation method from a constitutional point of view. Owing to globalisation and the cross-border activities of taxpayers, this issue is of central importance. Furthermore, taxpayers are entitled to invoke constitutional income taxation, and states are bound by a constitutional mandate to levy taxes in accordance with the constitution. States can orient the structure of their taxation on the quantitative income tax calculation methodology developed in this study to ensure constitutional income taxation for cross-border activities.

## 3 Quantitative analysis

If foreign income is received (AE) and the imputation method is applied (§ 34c EStG^3^), the tax base of the taxable income includes foreign income (AE) in addition to domestic income (IE). The basic tax-free allowance (GFB) is deducted from this sum within the framework of the respective tariff formula (§ 32a para. 1 EStG). The remaining amount after the deduction of the basic tax-free amount is multiplied by the effective income tax (tariff) rate for the total taxable income above the basic tax-free amount (S^g^).^4^ This results in the total income tax in the taxpayer's country of residence on the worldwide taxable income before the foreign tax credit (ESt_vor_34c_):

(1) $${ESt}_{vor\_34c}=\left(I_{E}+A_{E}-GFB\right)∙S^{g}$$

The income tax in the country of residence that is due on the foreign income (ESt__ auf_AE_) can be defined as follows, taking into account the basic allowance deduction (GFB):

(2) $${ESt}_{\_auf\_A_{E}}=\left(I_{E}+A_{E}-GFB\right)∙S^{g}∙\frac{A_{E}}{I_{E}+A_{E}}$$

(2.1) $${ESt}_{\_auf\_A_{E}}=\left[1-\frac{GFB}{I_{E}+A_{E}}\right]∙A_{E\;}∙S^{g}$$

(2.2) $${ESt}_{\_auf\_A_{E}}=\left[A_{E}-A_{E}∙\frac{GFB}{I_{E}+A_{E}}\right]∙S^{g}$$

The income tax in the country of residence that is due on domestic income (ESt__auf_IE_) can be shown accordingly, taking into account the basic allowance deduction (GFB):

(3) $${ESt}_{\_auf\_I_{E}}=\left(I_{E}+A_{E}-GFB\right)∙S^{g}∙\frac{I_{E}}{I_{E}+A_{E}}$$

(3.1) $${ESt}_{\_auf\_I_{E}}=\left[1-\frac{GFB}{I_{E}+A_{E}}\right]∙I_{E\;}∙S^{g}$$

(3.2) $${ESt}_{\_auf\_I_{E}}=\left[I_{E}-I_{E}∙\frac{GFB}{I_{E}+A_{E}}\right]∙S^{g}$$

Overall, the standard income tax in the country of residence on worldwide income before offsetting foreign taxes in accordance with § 34c EStG (ESt_vor_34c_) is therefore the sum of equations (3.2), (2.2):

(4) $${ESt}_{vor\_34c}=\left[I_{E}-I_{E}∙\frac{GFB}{I_{E}+A_{E}}\right]∙S^{g}+\left[A_{E}-A_{E}∙\frac{GFB}{I_{E}+A_{E}}\right]∙S^{g}$$

Equation (4) already shows that part of the basic tax allowance deduction (GFB) is taken into account when calculating the standard income tax in the country of residence, which is attributable to the foreign income that has already been subject to income tax in the foreign source country. Furthermore, the higher the share of foreign income in the worldwide income (IE + AE) or the higher the foreign income (AE) is compared with the taxpayer's domestic income (IE), the greater the share of the basic allowance deduction that is deducted when calculating the income tax in the country of residence that is due on the foreign income.

For example, if the foreign income (AE) is € 90,000 and the domestic income (IE) is € 10,000, the worldwide income is € 100,000. Thus, 90 % of the basic allowance deduction (GFB) affects the standard income tax of the country of residence, which is (proportionately) attributable to foreign income.

However, the (prorata) deduction of the basic tax-free allowance should not have any effect when determining the income tax in the country of residence that is attributable to foreign income if the crediting of foreign taxes (§ 34c EStG) is taken into account. The maximum amount of the foreign tax credit is equal to the amount of income tax in the country of residence applicable to the foreign income, whereby this maximum amount is represented by equation (2.2) or the second term in equation (4).

Furthermore, foreign income is subject to income tax in the foreign source state (S_A_). In the source state of foreign income, the taxpayer is generally subject to limited tax liability and is therefore taxed there on a gross basis; i.e., there is generally no basic tax allowance deduction in the foreign source state. The tax burden in the foreign source state with foreign income (S_A_) can be defined as follows, whereby s_α_ indicates the tax rate abroad:

(5) $$S_{A}=A_{E}∙s_{\alpha }$$

Taking into account the foreign tax credit in accordance with the imputation method (§ 34c EStG) in the taxpayer's country of residence, the following income tax burden results there (ESt_nach_34c_):

(6) $${ESt}_{nach\_34c}=\left[I_{E}-I_{E}∙\frac{GFB}{I_{E}+A_{E}}\right]∙S^{g}+\left[A_{E}-A_{E}∙\frac{GFB}{I_{E}+A_{E}}\right]∙S^{g}-\min\left\{\left[A_{E}-A_{E}∙\frac{GFB}{I_{E}+A_{E}}\right]∙S^{g}\;;\;\left(A_{E}∙s_{\alpha }\right)\right\}$$

The foreign tax credit is limited to the minimum of the standard income tax of the country of residence due on the foreign income and the foreign tax actually paid on the foreign income in the foreign source state.

If the tax burden on foreign income abroad is greater than or equal to the standard income tax in the taxpayer's country of residence payable on foreign income there, no standard income tax is charged on foreign income in the country of residence as part of the foreign tax credit (case 1). In this constellation, the deduction of the basic tax-free allowance has no effect on the taxpayer's country of residence insofar as its tax-reducing effect is attributable to the standard income tax in the country of residence, which is proportionately attributable to foreign income. Nevertheless, the standard income tax in the country of residence on foreign income is reduced to zero (€ 0) by offsetting foreign taxes. Consequently, the basic tax allowance deduction does not have a partial tax-reducing effect in the country of residence, although foreign income remains fully taxed, namely, at the tax burden in the foreign source country.

In this constellation (case 1), the following applies: Equation (5) > (2.2) or

$$S_{A}\geq {ESt}_{\_auf\_AE}\;;\;A_{E}∙s_{\alpha \;}\geq \left[A_{E}-A_{E}∙\frac{GFB}{I_{E}+A_{E}}\right]∙S^{g}$$

(7) $$s_{\alpha \;}\geq S^{g}∙\left[1-\frac{GFB}{I_{E}+A_{E}}\right]$$

Regarding case 1, the total tax burden on the taxpayer's income, taking into account the foreign tax credit in the country of residence and the deduction of the basic tax-free allowance (GFB), can be stated as follows (ESt_nach_34c_total_):

(8) $${ESt}_{nach\_34c\_total}=\left[I_{E}-I_{E}∙\frac{GFB}{I_{E}+A_{E}}\right]∙S^{g}+\left(A_{E}∙s_{\alpha }\right)$$

Formula (8) clearly shows that the basic allowance deduction (GFB) has only a partial tax-reducing effect in the taxpayer's country of residence, as it is partly attributable to the standard income tax in the country of residence, which is levied on foreign income but is reduced to zero (€ 0) because of the crediting of foreign taxes. As a result, the country of residence partially offsets the basic tax-free amount against fully taxed income, without this having a tax-reducing effect. The foreign income is taxed abroad with a foreign income tax, which, after offsetting foreign taxes or the tax reduction for foreign income, is equal to the standard income tax of the country of residence applicable to the foreign income (case 1).

In the case of taxable domestic income (IE), the basic tax allowance deduction (GFB) has only a partial tax-reducing effect in the ratio of domestic income to worldwide income (IE + AE). The greater the share of foreign income with a tax credit in the taxpayer's worldwide income or the lower the share of the taxable domestic income in worldwide income is, the less basic allowance deduction is proportionately attributable to the taxable domestic income.

As a result, the taxpayer in case 1 does not receive the full tax effect of the basic allowance deduction in the country of residence. Income in the amount of the basic tax-free allowance or the minimum subsistence level for tax purposes is not exempt from taxation with income tax in the country of residence. This is contrary to constitutional law and the requirements of the Constitutional Court, according to which income in the amount of the basic allowance deduction may not be subject to taxation (nontaxability).

The foreign income (AE) would have to amount to € 0, so that income in the amount of the basic tax-free allowance is effectively exempt from taxation. To achieve this, the pro rata deduction of the basic tax-free allowance in equation (8) for taxable domestic income would have to correspond exactly to the amount of the basic tax-free allowance. This would have to apply:

$$I_{E}∙\frac{GFB}{I_{E}+A_{E}}=GFB$$

$$I_{E\;}=GFB∙\frac{I_{E}+A_{E}}{GFB}$$

$$GFB∙\frac{I_{E}+A_{E}}{GFB}=I_{E}$$

$$A_{E}=0$$

The current tax calculation using the imputation method for foreign income (§ 34c EStG) means that part of the basic allowance deduction is offset against taxed foreign income and is not allocated to untaxed domestic income. A further effect of the current tax calculation method, in which part of the basic tax-free allowance is deducted when the standard income tax due on foreign income is calculated, is that fewer foreign taxes are available to offset. Furthermore, more income tax is due on taxable domestic income.

## 4 Numerical example and discussion

### 4.1 Example

The following example illustrates the potential unconstitutionality of the income tax calculation in the case of foreign tax credits (§ 34c EStG). The following data are assumed:

Single taxpayer/individual assessment.

Basic tax-free allowance (GFB): € 11,604 [income tax rate 2024 according to § 32a para. 1 EStG 2024 in the version of InflAusG].

Foreign income (AE): € 990,000; Domestic income (IE): € 10,000. Worldwide income (AE + IE = x) € 1,000,000.

Domestic effective tax rate (S^g^)^5^: 43.612 % [applicable tariff formula: 0.45x–18,936.88].

Foreign tax rate on foreign income (s_α_): 52 % [total foreign tax € 514,800^6^].

According to equation (6), the following applies to the tax burden in the country of residence, taking into account the foreign tax credit (§ 34c EStG):

$${ESt}_{nach\_34c}=\left[I_{E}-I_{E}∙\frac{GFB}{I_{E}+A_{E}}\right]∙S^{g}+\left[A_{E}-A_{E}∙\frac{GFB}{I_{E}+A_{E}}\right]∙S^{g}-\min\left\{\left[A_{E}-A_{E}∙\frac{GFB}{I_{E}+A_{E}}\right]∙S^{g}\;;\;\left(A_{E}∙s_{\alpha }\right)\right\}$$

Inserting the data, the following results are obtained:

$${ESt}_{nach\_34c}=\left[10.000-10.000∙\frac{11.604}{1.000.000}\right]∙0,43612+\left[990.000-990.000∙\frac{11.604}{1.000.000}\right]∙0,43612-426.748,67=€\;4,310$$

### 4.2 Discussion

The total income tax in the taxpayer's country of residence (ESt_nach_34c_) is € 4310. Owing to the current tax calculation, taking into account the imputation method for foreign income (§ 34c EStG), only 1 % of the basic tax-free amount of € 11,604 is deducted when calculating the standard tax due on taxable domestic income (I_E_), i.e., an amount of € 116.04. The remaining 99 % of the basic tax-free allowance of € 11,604, an amount of € 11,487.96, is deducted when the standard income tax due on foreign income (AE) is calculated. However, this part of the basic allowance deduction cannot have a tax-reducing effect because of the offsetting of foreign taxes. In this case (point 4.1), the offsetting of foreign taxes reduces the standard income tax in the country of residence, which is charged on foreign income, to zero (€ 0). As a result, the proportionate basic allowance deduction allocated to foreign income cannot have any tax effect in the country of residence. As a result, 99 % of the basic allowance deductions have no tax effect. Nevertheless, foreign income is fully taxed income.

In other words, the current tax calculation method using the imputation method for foreign income means that an amount of income equal to the minimum subsistence level or the basic tax-free allowance (€ 11,604) is not entirely exempt from income tax, as required by constitutional law. Instead, the basic tax-free allowance is (partially) offset against taxed foreign income and therefore cannot have the required tax-reducing effect in the taxpayer's country of residence.

## 5 Conclusion for the future organisation of taxation and tax law

### 5.1 Quantitative modelling of a constitutional tax system

The methodology for calculating income taxes, including the imputation method (foreign tax credit), should be changed so that it meets constitutional standards and thus does not violate the subjective net principle. To this end, the taxable minimum subsistence level in the form of income equal to the basic tax-free amount should be completely exempt from income tax.

Methodologically, constitutional taxation can generally be achieved if the basic tax-free amount (GFB) is deducted from taxable domestic income (IE) in the first step, insofar as domestic income is available.

There should no longer be a general proportional allocation of the basic exemption to domestic and foreign income according to their share of worldwide income, as is currently the case. Instead, there should be a direct allocation of the basic allowance deduction in the order mentioned here. If the basic tax-free allowance cannot be deducted from domestic income, it should be taken into account in a second step when calculating the tax on foreign income. Furthermore, a distinction should be made depending on whether the foreign tax on foreign income is higher than the standard rate of income tax levied on foreign income in the taxpayer's country of residence. This means that once foreign taxes have been credited, no additional income tax concerning the country of residence is due on foreign income. The same applies in the event that the standard rate of income tax in the taxpayer's country of residence on foreign income is higher than the income tax levied on foreign income in the foreign source state. This case means that in the taxpayer's country of residence, there is additional taxation of the foreign income over and above the taxation in the source country, with a residual income tax after offsetting the foreign tax.

Notably, even if the basic tax allowance deduction is allocated directly in the tax calculation in connection with foreign income, the basic tax allowance deduction may not have a (full) tax-reducing effect due to the offsetting of foreign taxes against the standard income tax of the country of residence attributable to the foreign income. This is particularly the case if the foreign tax burden on the foreign income is higher than the tax burden of the country of residence on the foreign income. In such a case, however, the foreign income is not untaxed. Rather, it is subject to a tax burden in the amount of the standard rate of income tax in the country of residence concerning the perspective of this state. In such a case, the country of residence and the country applying the imputation method for foreign income must reimburse the taxpayer an amount that corresponds to the tax effect of the basic tax-free amount attributable to the foreign income in the country of residence. Importantly, this is not a refund of foreign taxes by the taxpayer's country of residence but rather an exemption of the minimum subsistence level from taxation with income tax in accordance with the subjective net principle. By crediting foreign taxes in the amount of the domestic income tax due on foreign income, the country of residence recognises that foreign income is taxed in the same way as domestic income.

A constitutional calculation of income tax in the presence of foreign income, including the application of the imputation method, must take into account the exemption of the minimum subsistence level from taxation or the basic allowance deduction in accordance with the subjective net principle. This can be formulated as follows by equation (9):

$$\left(9.1\right){\;ESt}_{nach\_34c\_new}=\left[I_{E}-{{GFB}_{X}}^{\min\;\left(GFB,I_{E}\right)}\right]∙S^{g}-{\min\;\left[\left(GFB-{GFB}_{X}\right)∙S^{g}\;;\;\left[A_{E}-\left(GFB-{GFB}_{X}\right)\right]∙S^{g}\right]}_{Tax\;Refund}$$

$$if\;\left[A_{E}-\left(GFB-{GFB}_{X}\right)\right]∙S^{g}\leq \left(A_{E}∙s_{\alpha }\right)$$

$${\left(9.2\right)\;ESt}_{nach\_34c\_new}=\left[I_{E}-{{GFB}_{X}}^{\min\;\left(GFB,I_{E}\right)}\right]∙S^{g}+\left[A_{E}-{\left(GFB-{GFB}_{X}\right)}^{\min\;\left[\left(GFB-GFB_{x}\right);\;A_{E}\right]}\right]∙S^{g}-\left(A_{E}∙s_{\alpha }\right)$$

$$if\left[A_{E}-{\left(GFB-{GFB}_{X}\right)}^{\min\;\left[\left(GFB-GFB_{x}\right);\;A_{E}\right]}\right]∙S^{g}>\;\left(A_{E}∙s_{\alpha }\right)\;or\;A_{E}∙S^{g}>\left[\left(A_{E}∙s_{\alpha }\right)+{S^{g}∙\left(GFB-{GFB}_{X}\right)}^{\min\;\left[\left(GFB-GFB_{x}\right);\;A_{E}\right]}\right]$$

$$\left(9.3\right){\;ESt}_{nach\_34c\_new}=\left[I_{E}-{{GFB}_{X}}^{\min\;\left(GFB,I_{E}\right)}\right]∙S^{g}+{\left\{\left[A_{E}-{\left(GFB-{GFB}_{X}\right)}^{\min\left[\left(GFB-GFB_{x}\right);\;A_{E}\right]}\right]∙S^{g}-\left(A_{E}∙s_{\alpha }\right)\right\}}^{\geq 0}-{\left\{\left(A_{E}∙s_{\alpha }\right)∙\frac{{S^{g}∙\left(GFB-{GFB}_{X}\right)}^{\min\left[\left(GFB-GFB_{x}\right);\;A_{E}\right]}}{A_{E}∙S^{g}+{S^{g}∙\left(GFB-{GFB}_{X}\right)}^{\min\left[\left(GFB-GFB_{x}\right);\;A_{E}\right]}}\right\}}_{Tax\;Refund}$$

$$if\;A_{E}∙S^{g}<\left[\left(A_{E}∙s_{\alpha }\right)+{S^{g}∙\left(GFB-{GFB}_{X}\right)}^{\min\left[\left(GFB-GFB_{x}\right);\;A_{E}\right]}\right]\;and\;\left(A_{E}∙S^{g}-A_{E}∙s_{\alpha }\right)>0$$

Equation (9.1) for calculating income tax applies to the situation in which the foreign tax on the foreign income is higher than the income tax of the country of residence that is due there on the foreign income.

Equation (9.2) for calculating income tax applies to the situation in which the standard rate of income tax on the taxpayer's foreign income in the country of residence, including the tax effect of the basic tax allowance deduction, is greater than the foreign tax on the foreign income.

Equation (9.3) for calculating income tax applies to the situation in which the standard income tax on the taxpayer's foreign income in the country of residence is higher than the foreign tax on the foreign income without taking into account the tax effect of the basic tax allowance deduction but lower than the sum of the foreign tax on the foreign income and the tax effect of the basic tax allowance deduction, insofar as this is attributable to the foreign income.

The system of equations enables a constitutionally compliant calculation of income tax in the country of residence to be achieved when foreign income is received, and the imputation method is applied, taking into account the basic allowance deduction and compliance with the subjective net principle. Furthermore, it allows for the effective exemption of the minimum subsistence level from the income tax.

### 5.2 Application of the tax calculation equation (test) and discussion

This section presents a numerical example that is used to test the tax calculation equation (9). To this end, the numerical example from bullet points 4.1 and 4.2 is employed, wherein the complete exemption of the subsistence minimum is not granted according to the currently applicable tax calculation method. According to the currently applicable calculation method – see equation (6) – the tax-reducing basic allowance deduction is only granted in the amount of 1 % in this numerical example. The following data form the basis for the numerical example in point 4.1:

Single taxpayer/individual assessment.

Basic tax-free allowance (GFB): € 11,604 [income tax rate 2024 according to § 32a para. 1 EStG 2024 in the version of InflAusG].

Foreign income (AE): € 990,000.

Domestic income (IE): € 10,000.

Worldwide income (AE + IE = x): € 1,000,000.

Domestic effective tax rate (S^g^): 43.612 % [applicable tariff formula: 0.45x–18,936.88].

Foreign tax rate on foreign income (s_α_): 52 % [total foreign tax € 514,800].

In this taxation case, the foreign tax on foreign income (S_A_) is greater than the standard domestic income tax on foreign income (S^g^), including the deduction of the basic tax-free allowance (GFB). Consequently, equation (9.1) must be applied to calculate the income tax in the country of residence.

$$\left(9.1\right){\;ESt}_{nach\_34c\_new}=\left[I_{E}-{{GFB}_{X}}^{\min\;\left(GFB,I_{E}\right)}\right]∙S^{g}-{\min\;\left[\left(GFB-{GFB}_{X}\right)∙S^{g}\;;\;\left[A_{E}-\left(GFB-{GFB}_{X}\right)\right]∙S^{g}\right]}_{Tax\;Refund}$$

$$if\;\left[A_{E}-\left(GFB-{GFB}_{X}\right)\right]∙S^{g}\leq \left(A_{E}∙s_{\alpha }\right)$$

The aforementioned figures must be employed in the calculation of equation (9.1), with due consideration of the rules of the equation.

$$9.1){\;ESt}_{nach\_34c\_new}=\left[10.000-10.000\right]∙0,43612-{\min\;\left[\left(11.604-10.000\right)∙0,43612\;;\;\left[990.000-\left(11.604-10.000\right)\right]∙0,43612\right]}_{Tax\;Refund}$$

The result is:

(1) Consideration of basic tax-free allowance of € 10,000

Income tax on domestic income (IE) of € 10,000 = € 0→ at a tax rate of 43.612 %

(2) Consideration of basic tax-free allowance of € 1,604

Tax refund of € 1,604 × 0.43612 = € 699→ at a tax rate of 43.612 %

**Total basic tax-free allowance taken into account:€ 11,604** (100 %) **(1) + (2)**

and thus complete exemption of the minimum subsistence level from taxation.

With the current method of calculating income tax in the case of foreign tax credits (see points 4.1 and 4.2 and equation (6)) and the aforementioned numerical example, only 1 % of the basic allowance deduction (€ 11,604) is effectively granted. The exemption of the subsistence minimum from taxation with income tax is not achieved by the current tax calculation method. It therefore does not fulfil the requirements of constitutional law and the subjective net principle.

However, the situation is different with the income tax calculation method developed here, represented by equation (9). As the application of this calculation method shows, income equal to the basic allowance is effectively exempt from income tax in the country of residence. This means that the country of residence fulfils its obligation to exempt the taxpayer's minimum subsistence level from the income tax, even when using the credit method and receiving foreign income. Notably, in cross-border cases, the country of residence is obliged to grant the taxpayer the full amount of the basic deduction, i.e., to exempt from taxation income in the amount of the minimum subsistence level.

## 6 Summary and implications

This study addresses the question, which has not yet been raised and analysed, of whether the current method of calculating income tax in the case of foreign income and foreign tax credit violates constitutional law in the form of the subjective net principle. According to the subjective net principle, income up to the amount of the basic tax-free allowance, i.e., the taxpayer's minimum subsistence level, must be excluded from the income tax.

A quantitative analysis has demonstrated that the prevailing methodology for calculating income tax, coupled with the application of the imputation method (crediting of foreign taxes), infringes on the subjective net principle. This is because the taxpayer can lose part of the tax-effective basic tax allowance deduction in the country of residence under the current method of calculating income tax, meaning that their minimum subsistence level is no longer fully exempt from income tax.

The background to this is that under the current method of calculating income tax, part of the basic allowance deduction is offset against taxed foreign income and therefore does not have a tax-reducing effect in the country of residence under the imputation method for foreign taxes.

Nevertheless, the taxpayer's country of residence is constitutionally obliged to exempt the minimum subsistence level of the taxpayer from the income tax. It is therefore imperative that the country's tax regulations are amended to guarantee the complete exemption of the minimum subsistence level from the income tax. Those taxpayers who are affected can invoke the subjective net principle and constitutional law to claim the full tax-reducing effect of the basic tax allowance deduction. This study is therefore highly relevant because it concerns the fundamental taxation architecture of a state against the background of constitutional requirements and sets out implications for those taxpayers who are affected.

Moreover, a quantitative approach to calculating income tax was developed and tested (equations), which allows for a constitutionally compliant calculation of income tax in the country of residence when foreign income is received, and the imputation method is applied, taking into account the basic tax-free allowance deduction and compliance with the subjective net principle. It is therefore possible to guarantee, in principle, that the taxpayer's minimum subsistence level is exempt from income tax. Consequently, a change in the calculation of income tax is a viable option for states, which may choose to orient themselves on the approach developed here.

## Data availability statement

The data that support the findings of this study are available in the text of this article.

## Ethical approval

This article does not contain any studies with human participants or animals performed by any of the authors.

## Funding

No funds, grants, or other support was received. Open Access funding enabled and organized by Projekt DEAL.

## Declaration of competing interest

The author declares that he has no known competing financial interests or personal relationships that could have appeared to influence the work reported in this paper.

## Footnotes

1 For German income tax, the basic tax-free allowance is € 11,604 according to § 32a para. 1 no. 1 EStG 2024 in the version of InflAusG (German income tax act). This version of the tax tariff is used in this study. The deduction of this basic tax-free amount is included in all five tax formulas. In the case of splitting (spouses and joint assessment), the deduction of the basic tax-free allowance is doubled as a result. See § 32a para. 5 EStG.

2 See Germany, § 32a para. 1 EStG.

3 The crediting of foreign taxes in accordance with the German Income Tax Act (§ 34c EStG) is used as an example.

4 For the calculation and definition of S^g^, see also footnote 5.

5 Tax according to the tariff formula of § 32a EStG 2024 in the version of InflAusG with a taxable income of € 1,000,000 (0.45 × – 18,936.88) divided by taxable income above the basic tax-free allowance (1,000,000 – GFB). Here: (0.45 × 1,000,000–18,936.88): (1.000.000–11.604) = 0,43612.

6 990.000 € x 0,52 = 514.800 €.
